# *Talaromyces marneffei* infection and complicate manifestation of respiratory system in HIV-negative children

**DOI:** 10.1186/s12890-023-02390-y

**Published:** 2023-03-28

**Authors:** Qin Yang, Yue Wu, Xiaonan Li, Yanmin Bao, Wenjian Wang, Yuejie Zheng

**Affiliations:** 1 Department of Respiratory Diseases, Shenzhen Children’s Hospital, Shantou University Medical College, Shenzhen, 518038 China; 2 Department of Clinical Pharmacy, Shenzhen Children’s Hospital, Shantou University Medical College, Shenzhen, 518038 China

**Keywords:** *Talaromyces marneffei*, Respiratory system, Children, Antifungal therapy

## Abstract

**Background:**

Respiratory symptoms are the earliest clinical manifestation of *Talaromyces marneffei* (TM) infection. In this study, we aimed to improve the early identification of TM infection in human immunodeficiency virus (HIV)-negative children with respiratory symptoms as the first manifestation, analyze the risk factors, and provide evidence for diagnosis and treatment.

**Methods:**

We retrospectively analyzed six cases of HIV-negative children with respiratory system infection symptoms as the first presentation.

**Results:**

All subjects (100%) had cough and hepatosplenomegaly, and five subjects (83.3%) had a fever; other symptoms and signs included lymph node enlargement, rash, rales, wheezing, hoarseness, hemoptysis, anemia, and thrush. Additionally, 66.7% of the cases had underlying diseases (three had malnutrition, one had severe combined immune deficiency [SCID]). The most common coinfecting pathogen was *Pneumocystis jirovecii*, which occurred in two cases (33.3%), followed by one case of *Aspergillus sp.* (16.6%). Furthermore, the value of β-D-glucan detection (G test) increased in 50% of the cases, while the proportion of NK decreased in six cases (100%). Five children (83.3%) were confirmed to have the pathogenic genetic mutations. Three children (50%) were treated with amphotericin B, voriconazole, and itraconazole, respectively; three children (50%) were treated with voriconazole and itraconazole. All children were tested for itraconazole and voriconazole plasma concentrations throughout antifungal therapy. Two cases (33.3%) relapsed after drug withdrawal within 1 year, and the average duration of antifungal treatment for all children was 17.7 months.

**Conclusion:**

The first manifestation of TM infection in children is respiratory symptoms, which are nonspecific and easily misdiagnosed. When the effectiveness of anti-infection treatment is poor for recurrent respiratory tract infections, we must consider the condition with an opportunistic pathogen and attempt to identify the pathogen using various samples and detection methods to confirm the diagnosis. It is recommended the course for anti-TM disease be longer than one year for children with immune deficiency. Monitoring the blood concentration of antifungal drugs is important.

## Introduction

*Talaromyces marneffei* (TM) is an opportunistic, pathogenic, dimorphic fungus isolated from the liver of wild Rhizomyinae [1]. TM can invade the reticuloendothelial system, enter the blood circulation, and spread to other organs through the human respiratory and digestive tracts and prolonged skin contact, causing severe systemic infections, especially in immunosuppressed patients [2, 3]. TM is included to the first WHO fungal priority pathogens list (WHO FPPL) in 2022. The WHO FPPL focused on fungal pathogens that can cause invasive acute and subacute systemic fungal infections. It is developed to drive further research including paediatric patients [4]. TM infection in children is rare; however, it has a complex and insidious clinical presentation and a high mortality rate, mostly with respiratory tract infections as the first manifestation, most of which are accompanied by decreased numbers of T or Natural killer (NK) cells [5, 6]. Increasing studies have reported that TM infection in children should be considered as an important indicator of primary immunodeficiencies (PIDs) in human immunodeficiency virus (HIV)-negative individuals [7]. Therefore, fully understanding the clinical manifestations of TM infection and early initiating therapeutic measures is essential to improve the prognosis of the disease and to prevent mortality. In this study, the clinical features, genotype, treatment, and prognosis of six children were retrospectively analyzed to improve the understanding of the disease, identify risk factors early, and provide a basis for subsequent diagnosis, treatment, and prognostic assessment.

## Methods

### Patient data

The medical records of six patients who were diagnosed with TM infection between October 2017 and May 2022 at Shenzhen Children’s Hospital were reviewed; these HIV-negative children had respiratory symptoms as the first manifestation. The six patients were retrospectively analyzed for clinical presentation, underlying diseases, concurrent opportunistic pathogens, immune status, imaging and tracheoscopic findings, genetic results, treatment, and prognosis. This study has passed the ethical review of Shenzhen Children’s Hospital (201601306), which was conducted in accordance with the “Declaration of Helsinki” (revised in 2013). The written consent of each patient’s parents was obtained before participating in the study. They signed informed consent regarding publishing the data.

### ***Talaromyces marneffei*** examination

**Fig. 1:**
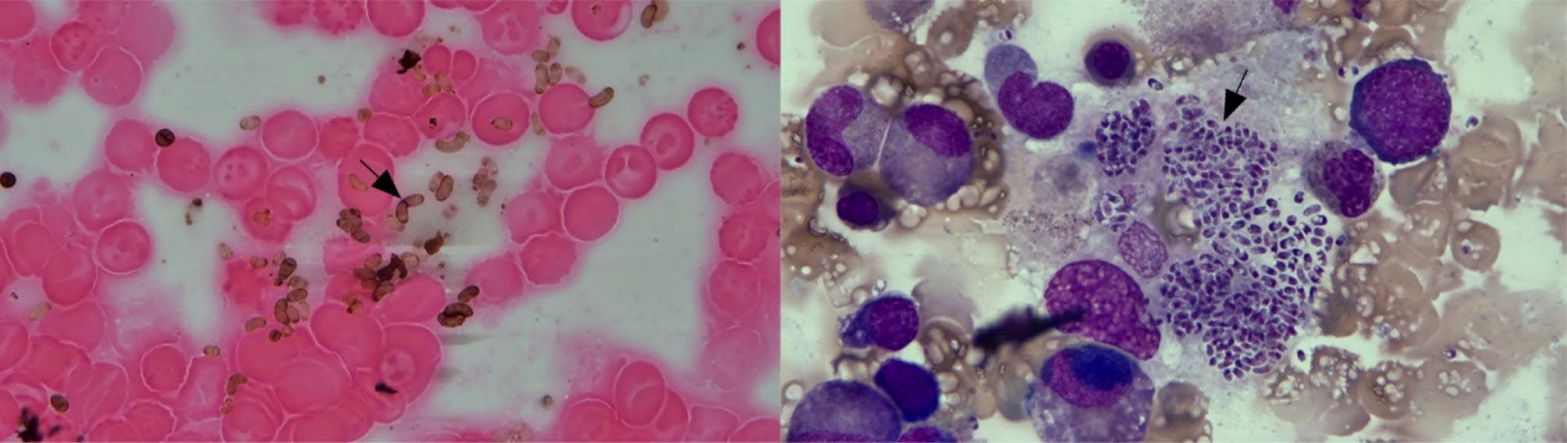
Bone marrow Gomori methenamine silver stain (left, oil immersion lens, 10 × 100 magnification) and Wright–Giemsa staining (right, oil immersion lens, 10 × 100 magnification) showing many *Talaromyces marneffei* cells outside the macrophages, which appeared sausage-like with two blunt ends, and part of the diaphragm was visible (black arrow)

Bronchoalveolar lavage fluid (BALF) and sputum specimens were inoculated in sterile Sabouraud agar medium in incubators at 25℃ and 37℃ to observe colony morphology, pigment production, and mycelial morphology using lactophenol phenol cotton blue staining. Blood and bone marrow specimens were incubated and monitored in a blood incubator, then observed via Gram staining or Gomori methenamine silver stain. Then, the samples were transferred and inoculated in Sabouraud agar medium at 25℃ and 37℃ in an incubator. The BALF, sputum, and tissue smears were then subjected to Gram and Wright–Giemsa staining, which showed that the bacterial cells were mulberry or grape bunch-like inside and outside the macrophages or blunt, round, and sausage-like at both ends, with the transverse septum visible in some parts. Furthermore, TM could be clearly diagnosed through culture isolation in the specimens, as shown in Fig. 1.

### (1,3)-β-D-glucan test (G test)

G test was used to measure serum (1,3)-β-D-glucan (BDG) level. 2 ml blood samples were collected after an overnight fast. The blood samples were centrifuged (3000 rpm, 10 min) within 0.5 h after collection. This assay required 20 µl of serum per sample according to manufacturer’s instructions (Dynamiker® Fungus (1,3)-β-D-Glucan assay, Dynamiker Biotechnology Co., Ltd, China). The cutoff value was 95 pg/ml (high cutoff threshold). BDG level between 70 and 95 pg/ml were considered indeterminate and level below 70 pg/ml (low cutoff threshold) were negative [8].

### **Gene** examination

Genomic DNA from peripheral blood samples was extracted following the manufacturer’s standard procedure using the QIAamp Blood Midi Kit (QIAGEN, Valencia, CA). Whole-exome sequencing developed by Agilent Technologies, Inc. (Santa Clara, USA) was adapted for the test. Briefly, an exome sequencing library of 1 µg genomic DNA was constructed using Agilent SureSelect Human All Exon v6 (Agilent Technologies Inc., Santa Clara, CA, USA) and then sequenced on Illumina NextSeq 500 platform (Illumina, San Diego, CA, USA). The generated 150-bp pairedend reads were pretreated by removing adapters and trimming the low-quality reads. The remained clean reads were aligned to the NCBI (National Center for Biotechnology Information) ‘s human reference genome (version hg38) using BWA software (version 0.7.17). Genome Analysis Toolkit (GATK) HaplotypeCaller function (version 4.1.2.0) was used to identify the variant sites. The variant interpretation was analyzed according to ACMG (American College of Medical Genetics and Genomics) criteria. The potential mutated sites were confirmed by Sanger sequencing on An ABI3730xl sequencer (Applied Biosystems, USA).

### Metagenome Next-generation sequencing (mNGS)

We collected 3 ml of BALF from each patient in cryogenic vials. The total nucleic acids of the BALF were extracted using enzymatic hydrolysis and bead milling methods. Then, the extracted nucleic acids were fragmented to approximately 200 bp via ultrasonication. The sequencing library was constructed via cyclization and the rolling circle replication of the fragments and then sequenced on a MGISEQ-200 high-throughput sequencing platform. Reads that mapped to human reference genomes or could be classified as low-complexity sequences were removed. The remaining data were then aligned to the Microbial Genome Databases retrieved from NCBI, consisting of viruses, bacteria, fungi, and parasites. The possible disease-causing pathogens were then identified from the sequencing results.

### Antifungal plasma concentration monitoring

Voriconazole (VRZ): At least 1 ml blood samples of steady-state trough VRZ concentrations were collected for the enzyme multiplied immunoassay technique (EMIT) method. The blood samples were centrifuged (3000 rpm, 8 min) within 24 h after collection. A 4 µl aliquot of each plasma sample was determined by the EMIT analysis in Shenzhen Children’s Hospital. The procedures were performed according to the VRZ assay kit protocol (No.20190401, Zhuhai Lizhu Reagent Co., Ltd). Briefly, blood samples were collected in EDTA anticoagulant tubes, centrifuged at 3000 rpm for 8 min, and 4 µl of supernatant was analysed on a Viva-E automatic drug concentration analyser (Siemens, Germany). A calibration curve was constructed with the following VRZ concentrations: 0.0, 0.5, 1.5, 4.0, 8.0, and 16.0 µg/ml (using working standard solution). Three-level controls (1.0 µg/ml as low-L; 5.0 µg/ml as medium-M; 10.0 µg/ml as high-H) were used for daily quality control (QC). The variation coefficients of these EMIT-measured controls were 14.9%, 10.7%, and 10.9%, respectively. QC concentrations were then prepared at 1.0, 5.0, and 10.0 µg/ml. As recommended by guidelines [9], the effective VRZ concentration range is 1.0–5.0 µg/ml.

Itraconazole concentration was determined by Waters Acquity UPLC I-Class IVD/ Xevo TQ-D IVD liquid chromatographer-tandem mass spectrometry. For the detection of plasma concentration, 100 µL of plasma sample was placed in 1.5 mL EP tube, 300 µL of the internal standard itraconazole-D9 solution was added, followed by rapid vortex oscillation for 1 min, centrifugation at 14,000 rpm/min at 4℃ for 10 min, and then 200 µL of supernatant was drawn for sampling.

## Results

### Demographic characteristics and clinical manifestations

The age of disease onset of six children ranged from 8 months to 8 years (4.2 ± 3.8 years). There were five males (83.3%) and one female (16.7%). Respiratory system infection was the first manifestation in all cases; all cases (100%) had cough and hepatosplenomegaly, and most patients (83.3%) had a fever. Out of the six cases, lymph node enlargement was noted in three cases (50%), and two cases (33.3%) had a rash. Rales could be heard in two cases (33.3%), and wheezing and hoarseness were present in one patient (16.6%). Furthermore, hemoptysis and anemia occurred in one case (16.6%), and one case (16.6%) had thrush.

Of all the cases, 66.7% had underlying diseases (three patients had malnutrition, and one had severe combined immune deficiency [SCID]). Specifically, P1 had a history of tuberculosis of the spleen, varicella, and recurrent respiratory infections (RRTIs), and P2 had a previous diagnosis of an axillary abscess, RRTI, and *Salmonella typhimurium* infection. The most common coinfecting pathogen was *Pneumocystis jirovecii*, which occurred in two cases (33.3%), followed by one case (16.6%) of *Aspergillus sp.*. The duration from symptom onset to diagnosis ranged from 1 to 3 months in all six children.

The diagnosis of TM infection relied on a variety of samples and detection methods, including a sputum smear and culture (16.7%), lung tissue (16.7%) and lymph node tissue (16.7%) biopsy, blood culture (33.3%), BALF culture (16.7%), bone marrow culture (16.7%), and BALF (16.7%) and blood (16.7%) mNGS, individually or in combination (Table 1; Fig. 1).

Pathogenic variants were detected through Whole-exome sequencing of the six children. Of these, five children (83.3%, P1–P5) had confirmed PID. P6, with a hemizygote variation at the related site of the *CD40LG* gene, had uncertain pathogenicity. Additionally, three cases (50%) had *STAT1* variations, one case (16.7%) had a large intragenic deletion variation of the *CD40LG* gene, and one case (16.7%) had a hemizygous variation of *IL2RG.*

**Table 1:** Demographic characteristics and clinical data of children with *Talaromyces marneffei* infection

Patient	Sex	Age (years)	Time from onset to diagnosis	Clinical manifestation	Affected organs	Family history	Base diseases	Medical history	Mutant gene	Concurrent opportunistic pathogens	Pathogenic confirmed specimens
**P1**	Female	8	1 month	Fever, cough, enlarged lymph nodes, enlarged liver and spleen, rash	lung, liver, spleen, lymph node, and skin	None	Mild malnutrition	Splenic tuberculosis, varicella, RRTI	*STAT1*	None	Lung tissue biopsy culture
**P2**	Male	1.2	3 months	Fever, cough, enlarged liver and spleen	lung, liver, and spleen	None	Mild malnutrition	Axillary abscess, RRTI, *Salmonella typhimurium* infection	*CD40LG*	None	Blood culture
**P3**	Male	8.7	2 months	Fever, cough, hemoptysis, enlarged lymph nodes, enlarged liver and spleen	respiratory tract, liver, spleen, lymph node	None	Moderate malnutrition	None	*STAT1*	None	Lymph node biopsy tissue culture, BALF mNGS
**P4**	Male	1.3	1 month	Wet cough, wheezing, hoarseness, shortness of breath, wet rales, enlarged liver and spleen	respiratory system, liver, and spleen	None	None	None	*STAT1*	*Aspergillus sp., Pneumocystis jirovecii*	BALF mNGS, BALF smear microscopic exam
**P5**	Male	0.67	20 days	Fever, cough, anemia, rash, enlarged liver and spleen, hematuria	lung, liver and spleen, skin, urinary system	Brother died of TM infection	SCID	None	*IL2RG*	*Pneumocystis jirovecii*	Blood culture, sputum culture, medullo culture
**P6**	Male	6	20 days	Fever, cough, enlarged cervical lymph nodes, thrush, enlarged liver and spleen, moist rales, hemophagocytic lymphohistiocytosis	lung, liver, spleen, lymph node, and blood system	None	None	None	Uncertain	None	Blood smear, blood culture, lymph node biopsy, BALF, blood mNGS

- negative, + positive; SCID: Severe combined immune deficiency; RRTI: Recurrent respiratory tract infection; BALF: Bronchoalveolar lavage fluid; mNGS: metagenome Next-generation sequencing; TM: *Talaromyces marneffei*

## Laboratory examination

We detected elevated (1,3)-β-D-glucan (G test) in three cases (50%, P1, P2, P3). Additionally, the IgG titer was increased in three cases (50%), while two cases (33%, P2, P5) had decreased IgA, two cases (33%, P2, P5) had decreased IgM, and complement C3 and C4 levels were normal. Six children (100%) had decreased NK cell ratios. P5 was diagnosed with SCID and had significantly decreased total T, CD4 + T, CD8 + T, and NK cells (Table 2).

**Table 2:** Results of laboratory examinations of children with *T. marneffei* infection

	P1	P2	P3	P4	P5	P6
G test (0–70.0 pg/ml)	74.51	815.8	115.93	< 37.5	< 37.5	Unchecked
IgA (g/l)	2.83 (0.51–2.97)	0.09 (0.19–2.22)	3.70 (0.51–2.97)	1.3 (0.41–2.97)	0.01 (0.19–2.22)	0.94 (0.41–2.97)
IgG (g/l)	34.76 (5.28–21.9)	21.83 (2.86–16.8)	24.36 (5.28–21.9)	14.06 (5.28–21.9)	2.32 (2.86–16.8)	7.75 (5.28–21.9)
IgM (g/l)	1.22 (0.48–2.26)	0.38 (0.43~)	1.09 (0.48–2.26)	1.64 (0.48–2.26)	0.02 (0.43 ~ 1.63)	0.64 (0.48–2.26)
IgE (IU/l)	9.57	0.1	13.8	2.12	Unchecked	Unchecked
C3 (g/l)	1.2 (0.7–2.06)	1.64 (0.7–2.06)	1.55 (0.7–2.06)	1.38 (0.7–2.06)	0.77 (0.7–2.06)	0.78 (0.7–2.06)
C4 (g/l)	0.12 (0.11 ~ 0.61)	0.26 (0.11 ~ 0.61)	0.44 (0.11 ~ 0.61)	0.31 (0.11–0.61)	0.22 (0.11 ~ 0.61)	0.15 (0.11–0.61)
Total T-lymphocytes (%)	67.25 (56–86)	80.87 (56–86)	76.74 (57.1–73.43)	55.68 (53.88–72.87)	6.03 (55.32–73.11)	52.29 (60.05–74.08)
CD4 + T cells (%)	42.06 (33–58)	54.88 (33–58)	33.77 (33–58)	49.1 (33–58)	1.63 (33–58)	27.32 (33–58)
CD8 + T cells (%)	19.28 (13–39)	23.18 (13–39)	25.48 (13–39)	20.97 (13–39)	4.46 (13–39)	23.47 (13–39)
NK cells (%)	3.52 (5–26)	1.96 (5–26)	3.45 (5–26)	1.3 (5–26)	1.87 (5–26)	2.07 (5–26)
B cells (%)	27.12 (5–22)	15.40 (5–22)	14.06 (5–22)	25.42 (5–22)	91.46 (5–22)	41.85 (5–22)

G test (0–70.0 pg/ml): G test reference values according to Dynamiker® Fungus (1–3)-*β*-D-glucan assay

### Chest computed tomography and tracheoscopic manifestations

**Fig. 2:**
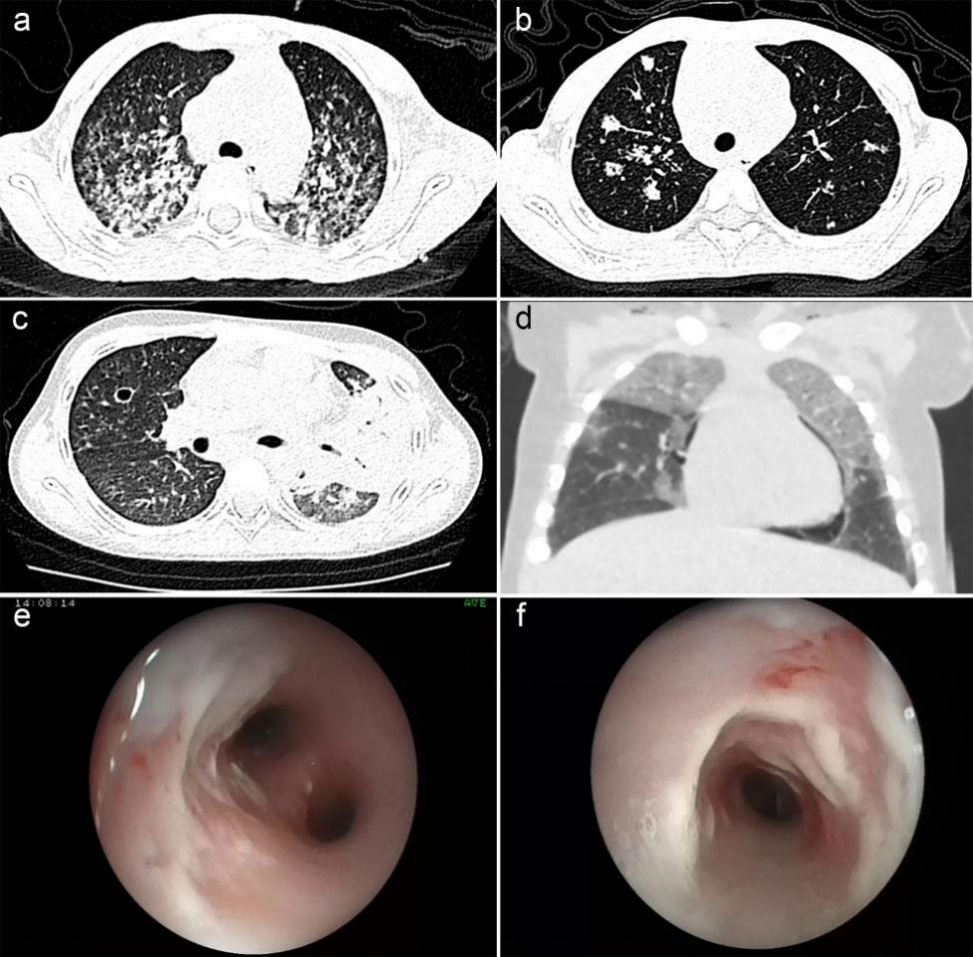
High-resolution computed tomography and tracheoscopic images show: (a) interstitial infiltration in both lungs, (b) distribution of multiple masses in the distal bronchus, (c) large consolidation of the left lung lobe with the pulmonary cavity, (d) mediastinal emphysema, (e, f) mucosal edema and erosion, and a large number of white adhesions in the intima of the trachea

Computed tomography (CT) scan revealed mediastinal lymphadenectasis (66.6%, P1, P3, P4, P6), nodular shadow (50%, P1, P3, P4), consolidation of the lung (33.3%, P1, P5), interstitial lung disease (16.7%, P2), mediastinal pneumoperitoneum (16.7%, P5), pleural effusion (16.7%, P2), and the pulmonary cavity (16.7%, P1). Furthermore, five cases (83.3%, P1–P4, P6) had a white secretion under the tracheoscopy, and two patients (33.3%, P3 and P4) had a bean curd residue-like tracheal secretion (Fig. 2).

## Antifungal therapy and outcome

Two cases (P2, P5) did not complete follow-up, and four cases (66.7%, P1, P3, P4, P6) improved. Two patients (33.3%, P1, P2) relapsed after 6 months and 9 months of antifungal treatment, respectively. The average duration of antifungal treatment in the children was 17.7 months (6–51 months). The plasma concentrations of voriconazole or itraconazole were detected to determine the appropriate dosing in all cases. Three children (50%, P1, P5, P6) were treated with amphotericin B, voriconazole, and itraconazole, respectively. P1 received voriconazole (100-175 mg twice daily) for 6 months, and her cough recurred after withdrawal after 5 months, recurrence was considered. Antifungal treatment was then restarted for 6 months. However, CT scan indicated that her pulmonary lesions had worsened, and the liver function test was abnormal. Finally, amphotericin B was administered intravenously for 1 month, and itraconazole (5 mg/kg twice a day) was taken orally for 44 months. Of note, this girl had been on antifungal therapy for more than 4 years. P5, in the initial stage of his disease, the causative agent had not been identified and as routine antifungal treatment, amphotericin B was administered intravenously for 1 week and oral voriconazole (50 mg twice daily) for 5 months. The disease progressed until the pathogen was identified. When his symptoms improved, amphotericin B was administered intravenously for 1 week, and itraconazole (5-6 mg/kg twice a day) was administered orally for 6 months. His parents requested automatic discharge, so he did not complete follow-up. Moreover, P6 received amphotericin B and voriconazole injections for 1 month and 18 days, respectively; after his symptoms improved, his parents requested that the traditional voriconazole tablets be replaced by an itraconazole oral that was easier to swallow and measure doses. Itraconazole (3-4 mg/kg twice daily) was taken orally for 12 months, and he continues to receive antifungal treatment.

Three children (50%, P2, P3, P4) were treated with voriconazole and itraconazole. P2, who had not clinically improved after receiving voriconazole for 10 days, changed to itraconazole intravenously for 2 weeks, then oral itraconazole (3-4 mg/kg twice daily) for 12 months. Additionally, intermittent antifungal therapy was provided for 1 year, during which time there was recurrence. This patient did complete follow-up. For P3, voriconazole was administered intravenously for 10 days. After his symptoms improved, he was switched to oral voriconazole, but it caused nausea and headache. Oral itraconazole (5 mg/kg twice a day) was then given for 12 months, and antifungal drugs were administered for more than 1 year. For P4, voriconazole was given intravenously for 3 days until the pathogen was identified and switched to oral itraconazole at a dose of 5 mg/kg 3 times daily for 3 days, followed by twice daily, then maintained at 6.5 mg/kg twice daily based on the concentration of itraconazole. After 2 weeks, his symptoms improved significantly. Notably, itraconazole had been administered for nearly 1 year (Table 3).

**Table 3:** Treatment administered to children with *Talaromyces marneffei* infections

Patient	Antifungal therapy	Treatment of combined infection and underlying diseases	Antifungal course (month)	Outcome
P1	voriconazole + amphotericin B + itraconazole	None	51	Improved
P2	voriconazole + itraconazole	None	12	No follow-up
P3	voriconazole + itraconazole	None	12	Improved
P4	voriconazole + itraconazole	Compound sulfamethoxazole	12	Improved
P5	amphotericin B + voriconazole + itraconazole	Regular intravenous Immunoglobulin, Compound sulfamethoxazole	6	No follow-up
P6	amphotericin B + voriconazole + itraconazole	Compound sulfamethoxazole, hemophagocytic lymphohistiocytosis treatment	12	Improved

## Discussion

TM is an important thermally dimorphic fungus known to be pathogenic in mammals (including humans) [10]. Immunocompetent populations can be infected with TM; however, infections are most common in patients with defective CD4 + T-cell activity [11]. The lungs are the main target organ of this pathogenic fungus, which can spread to other organs via lymphatic or hematogenous dissemination and progress rapidly, with a morbidity and mortality rate of up to 20% if antifungal therapy is not administered promptly [12]. Therefore, the respiratory system is the “window” through which TM spreads to the whole body. Currently, there are few systematic analyses of the risk factors and vulnerability of HIV-negative children to TM infection. Furthermore, the clinical features in children with TM infection are nonspecific, meaning that they are not easily identified and can lead to misdiagnosis and underdiagnosis[13, 14]. Currently, there are no standard treatments for TM infection. Therefore, this study reviewed and analyzed the clinical manifestations of TM infection in HIV-negative children, with the respiratory system as the first location of symptoms, and analyzed the risk factors associated with infection.

The children with TM infection in this study were mainly males, with the youngest age of infection being 8 months. All the children had cough and hepatosplenomegaly, and fever was the most common symptom. Moreover, two cases had hoarseness, wheezing, and hemoptysis separately when the infection involved the pharynx and airway. These features were consistent with previous reports [15, 16]. Furthermore, TM infection involved the extra-pulmonary organs, including bone, skin, lymph nodes, and/or the central nervous system [17]. In this study, one-half of the children had enlarged lymph nodes, and one-third of them developed a rash. The clinical manifestations of non-HIV children with TM infection differ from those in non-HIV adults. Fever and spleen enlargement were less common in adults, but they are more likely to have bone destruction and skin lesions, including rashes, nodules, abscesses, and ulcers [18, 19]. However, no signs of bone destruction were found in this study, and only 2 cases showed rash. Children with TM infection are more likely to have hemophagocytic lymphohistiocytosis (HLH), but this condition is rare in adults [20, 21]. In this study, one child developed HLH. In general, the clinical manifestations of non-HIV children with TM showed no significant specificity, which was easily misdiagnosed, leading to delayed treatment and the occurrence of HLH. Meanwhile, it was related to the abnormal immune function of children. In this study, 5 children showed gene mutations corresponding to different primary immunodeficiency diseases, resulting in the dysfunction of TM clearance and abnormal immune response, leading to severe manifestations of HLH [20]. Therefore, TM infection should be highly thought of when non-HIV children combined with PID have fever, cough, lymph node enlargement, and even HLH, with poor anti-infection effect.

Previous studies have confirmed that inhalation is the main mode of TM invasion into the body, and the lung is the most important organ for TM infection [22]. Furthermore, TM avoids phagocytosis of macrophages by adhering to bronchial epithelial cells via adhesion to the extracellular matrix while producing superoxide dismutase and peroxidase to prevent digestion by lysosomes [23]. However, engulfed TM proliferates within macrophages and activates the release of inflammatory cytokines (IL-1β, IL-6, IL-8, IL-10, IL-12, IL-16, IL-18, TGF-β, TNF-α) and chemokines (IP-10), which play an important role in defending against TM infection and propagate through the reticuloendothelial system, causing lymphadenectasis and multi-organ damage. The cytokines and chemokines recruit more neutrophils and monocytes to the site of infection, stimulating the antifungal effector functions of macrophages and neutrophils to defend against TM infection [24–27]. Too many inflammatory cytokines can trigger a cytokine “waterfall effect” that results in oxidative stress, which might contribute to disease severity and poor clinical outcomes [25]. In this study, P6 developed phagocytic syndrome due to the strong inflammatory response experienced after infection, which was considered to be related to the inflammatory “waterfall effect” produced by TM-mediated activation of inflammatory cytokines [27].

In this study, the median time from illness onset to diagnosis was 41 days (range: 20 days to 3 months), which was related to the atypical clinical manifestations that could easily be misdiagnosed as RRTI, leading to a delay in diagnosis. For example, P1 was initially diagnosed with pulmonary tuberculosis due to multiple nodular shadows and diffuse lesions appearing on her chest CT scan. The literature suggests that 31.1% of HIV-negative patients with TM infection are misdiagnosed with tuberculosis and receive long-term antituberculosis treatment but have poor treatment outcomes [5]. This study demonstrated that classical diagnostic principles for the diagnosis of TM are fungal culture or histopathological examination [28]; five children were diagnosed through fungal culture and/or biopsy culture in this study. P4 had recurrent cough and wheezing and showed no response to the antibiotic, and TM was eventually confirmed via BALF smear microscopy and mNGS. Therefore, it is recommended that, in a child with respiratory tract infection as the first manifestation whose pathogen is difficult to identify, various sample types (sputum, blood, BALF, tissue biopsy) and different detection methods (smear, culture, mNGS) should be used to make up for the limitations of a single technique or sample.

G test, used for detecting and semi-quantifying β-glucan content during fungal preparation, was reported to be elevated in 50% of patients. Moreover, P5 had severe immunodeficiency and an overall decrease in immunoglobulin and lymphocyte ratios, while the remaining five children had no significant abnormalities in immunoglobulin, complement, or lymphocyte counts. However, all children showed a decrease in the proportion of NK cells. Previous studies have found that HIV-negative patients with TM infection may experience a temporary decrease in NK cells due to consumption during the infection, which can be restored with the improvement of the condition [27, 29]. Normal lymphocyte counts do not represent normal immunity, as cell function may be abnormal. Therefore, further assessment of lymphocyte function is necessary to understand the immune status of patients [27].

Notably, two cases had opportunistic pathogens coinfection, and the most common pathogen was *P. jirovecii*, followed by *Aspergillus sp.*. Previous studies have shown that patients with HIV/acquired immunodeficiency syndrome (AIDS) are susceptible to infection with TM, which also has an increased incidence in patients with other immune defects. *P. jirovecii* is one of the most common pathogens in immunosuppressed hosts, and mixed mycosis in HIV-negative patients with immunosuppression is not uncommon [17, 30]. To elucidate TM infection or mixed mycosis that occurred in the HIV-negative children, genetic tests were performed for all children in this study. Five children had the pathogenic variants, and the mutated genes were *STAT1*, *CD40LG*, and *IL2RG*. Mutations in *STAT1*, *CD40LG*, and *IL2RG* correspond to different immunodeficiency diseases, following number and function abnormality of the natural immune and adaptive immune cells, leading to these patients being susceptible to the fungus. 87.8% of the 90 patients with *STAT1* mutation showed Th17 cytopenia [31]. This mutation can decrease IL-17 secretion through two mechanisms, (1) directly inhibits the differentiation of T cells into Th17 cells; (2) impairs the pathway that IL-6, IL-21, and IL-23 induce Th17 cell differentiation through *STAT3* [32]. The decreased Th17 differentiation impairs IL-17 function in the defense against extracellular pathogens like fungi, which might explain the patient’s susceptibility to *T.marneffei* [32, 33]. *CD40LG* mutation can cause X-linked hyper-immunoglobulin M (XHIGM), which has been classified as combined T and B immunodeficiency [34]. A hemizygous mutation of *IL2RG* was identified in one patient diagnosed with SCID [35]. The number and function of T and NK cells were absent in children with SCID, severely reducing the body’s antifungal immune part. XHIGM and SCID are primary immunodeficiency diseases (PID). According to previous reports, patients with PID are more susceptible to *T. marneffei* [36]. Therefore, TM should be considered an “early warning sign” for immunodeficiency disease in HIV-negative children [37].

The imaging characteristics of TM infection are nonspecific. Specifically, mediastinal lymph node enlargement was the most common finding in this study. Nodular shadows, consolidation, interstitial infiltration, cavities, mediastinal emphysema, and pleural effusion were also observed, which is consistent with previous reports [38]. Additionally, white secretions could be seen with a bronchoscope.

Currently, there is no standard for treating TM infection in HIV-negative patients. Current treatment is based on studies of TM infection in HIV-positive patients, with long-term consolidation and maintenance treatment with itraconazole after 2 weeks of highly effective amphotericin B induction therapy [20]. Voriconazole and itraconazole are also used in the treatment of TM in non-HIV patients, and their safety and efficacy have been confirmed [20, 37, 38]. However, amphotericin B can cause serious adverse effects, such as liver and kidney damage and severe hypokalemia [39]. Therefore, the ability of children to tolerate the drug should be considered. In our study, amphotericin B was mainly used in children with recurrences and severe disease onset at the initial stage. Two children relapsed after stopping antifungal therapy within one year, and the entire antifungal treatment course in all children was much longer than the duration recommended for adults. Therefore, we speculate that there is persistent fungal susceptibility caused by PID [40]. All the children repeatedly monitored the plasma concentration of antifungal drugs to determine the dose. Excluding the two children who did not complete follow-up, all the remaining children improved. Therefore, antifungal therapy should be personalized and more than one year for immunodeficient children with respiratory tract TM infection. Furthermore, the global response should be assessed at the end of treatment based on the overall clinical, mycological, radiological, and immune state [41].

## Conclusion

TM infections in children mostly have respiratory symptoms as their first manifestation. TM infection should be considered if a child presents with cough, fever, hepatosplenomegaly, and lymphadenectasis that does not respond to antibiotic therapy. It is necessary to employ different methods, including sputum smears, cultures, tissue biopsies, and mNGS from various samples (blood, bone marrow, BALF, lymph nodes, or lung) to determine the pathogen and any coinfections. In addition, clinicians should not view TM infection as the endpoint of diagnosis and need to inquire about clinical manifestations related to immunodeficiency and discover any underlying immunodeficiency diseases. Nevertheless, the duration of anti-TM therapy and the indicators for discontinuation are currently unclear in HIV-negative children. In this study, patients were prone to relapse due to the cessation of antifungal treatment within one year. Therefore, it is recommended that the treatment time for anti-TM infection be longer than one year, especially for patients with immunodeficiency. Antifungal drug concentrations must be monitored throughout the treatment period.

## Data Availability

The datasets used and/or analyzed during the current study are available from the corresponding author on reasonable request.

## Abbreviations

TM: Talaromyces marneffei
HIV: human immunodeficiency virus
SCID: severe combined immune deficiency
NK: Natural killer
PIDs: primary immunodeficiencies
BALF: Bronchoalveolar lavage fluid
mNGS: metagenome Next-generation sequencing
VRZ: Voriconazole
EMIT: enzyme multiplied immunoassay technique
QC: quality control
RRTI: Recurrent respiratory tract infection
CT: Computed tomography.
